# How Can We Reduce Prolonged Mental Health Admissions to Paediatric Wards, at Sheffield Children’s Hospital?

**DOI:** 10.1192/bjo.2026.11608

**Published:** 2026-06-30

**Authors:** Rianne Parmar, Russ Birkett, Fran Porter-Young

**Affiliations:** Sheffield Children's Hospital, Sheffield, United Kingdom

## Abstract

**Aims:**

To understand what leads young people to come to Sheffield Children’s Hospital for their mental health, and the barriers to discharge home. This may help guide the service in reducing the number of long admissions and their length of stay. For example, we may be able to identify at risk groups and give appropriate support before and during admission.

**Methods:**

We gathered data showing admissions to the STAR team (Supportive Treatment and Recovery Team) from 2022-2024 and identified patients with a prolonged (>21 days) stay. Within their electronic record, notes were reviewed to identify: patient demographics, frequency of stays (related to mental health), length of stays (related to mental health), risk to self or others, involvement of other services or consideration of Tier 4 input, issues with sleep, school, substances, possible/confirmed neurodiversity.

**Results:**

Out of 753 patients identified in 2022-2024, 20 patients met the criteria for a prolonged admission. There was an even split by sex, with a mean age of 13.5 years. We identified demographic traits within the cohort having prolonged admissions.

**Conclusion:**

Overall, it seems certain factors might increase the risk of a prolonged mental health admission, such as safeguarding concerns and violence and absconding risk. Using the risk factors identified, we have created an admissions tool. The aim is for this to allow professionals to score the amount of risk factors, and if meeting a certain threshold, trigger a case conference. This may help at the early stages to reduced potential frequent or prolonged admissions for a young person.

